# Molecular Identification of Selected Tick-Borne Protozoan and Bacterial Pathogens in Thoroughbred Racehorses in Cavite, Philippines

**DOI:** 10.3390/pathogens10101318

**Published:** 2021-10-13

**Authors:** Eloiza May Galon, Adrian Miki Macalanda, Mary Margarett Garcia, Chrysler James Ibasco, Anatolio Garvida, Shengwei Ji, Iqra Zafar, Yae Hasegawa, Mingming Liu, Rochelle Haidee Ybañez, Rika Umemiya-Shirafuji, Adrian Ybañez, Florencia Claveria, Xuenan Xuan

**Affiliations:** 1 National Research Center for Protozoan Diseases, Obihiro University of Agriculture and Veterinary Medicine, Obihiro 080-8555, Japan; eloizagalon@gmail.com (E.M.G.); jishengwei0903@hotmail.com (S.J.); eekrawahla@hotmail.com (I.Z.); y13665trp@gmail.com (Y.H.); umemiya@obihiro.ac.jp (R.U.-S.); 2 Department of Immunopathology and Microbiology, College of Veterinary Medicine and Biomedical Sciences, Cavite State University, Indang 4122, Philippines; marymargarettfgarcia@gmail.com (M.M.G.); chryslerjamesibasco@gmail.com (C.J.I.); 3 Saddle & Clubs Leisure Park, Philippine Racing Club Inc., Naic 4110, Philippines; tolygarvida@gmail.com; 4 Department of Microbiology and Immunology, School of Basic Medicine, Hubei University of Arts and Science, Xiangyang 441053, China; lmm_2010@hotmail.com; 5 Institute of Molecular Parasitology and Protozoan Diseases, Main Campus and College of Veterinary Medicine, Barili Campus, Cebu Technological University, Cebu City 6000, Philippines; rochelledybanez@gmail.com (R.H.Y.); dr.adrianpybanez@gmail.com (A.Y.); 6 Biology Department, College of Science, De La Salle University, Manila 1004, Philippines; florencia.claveria@dlsu.edu.ph

**Keywords:** *Anaplasma*, *Babesia*, *Borrelia*, *Theileria*, horse, Philippines

## Abstract

Tick-borne diseases (TBDs) considerably impair equine health and productivity. Moreover, TBDs, particularly equine piroplasmosis, impede international movement and trade of equids, which is a vital component of the global horse racing industry. In the Philippines, horse racing is a lucrative industry generating millions of USD annually. However, information on equine TBDs is scarce. This study intended to describe molecularly the equine tick-borne infections in a racehorse park in Cavite, Philippines and identify the risk factors associated with the infections. One hundred twenty-four (n = 124) thoroughbred racehorses were sampled and screened for selected tick-borne protozoan and bacterial pathogens using polymerase chain reaction (PCR) assays. Racehorses were positive for *Babesia caballi* (12.10%; 15/124), *Theileria equi* (0.81%; 1/124), *Anaplasma phagocytophilum* (10.48%; 13/124), *Borrelia burgdorferi* sensu lato (38.71%; 48/124), *A. marginale* (0.81%; 1/124), and *Coxiella burnetii* (0.81%; 1/124). *Rickettsia* was not detected in the samples. Gender was determined as a significant risk factor for *B. caballi* infection. Sequencing analysis revealed that seven partial 18S rRNA *B. caballi* isolates shared 98.63–100% identity with each other and were classified as genotype A. Meanwhile, the sequence obtained from the lone *T. equi*-positive sample was 99.77% identical to isolates from Spain, Switzerland, China, Saudi Arabia, and South Korea, and was confirmed as genotype E based on the 18S rRNA gene. Eight *Anaplasma* 16S rRNA partial sequences were highly identical to *A. phagocytophilum* and *A. ovis*. Partial sequences of *Borrelia* 5–23S rRNA were most closely related to *B. japonica* and other *Borrelia* sp. isolates from various countries. This study reports the first molecular detection of *Borrelia* and *Anaplasma* and the identification of *B. caballi* and *T. equi* genotypes in racehorses in the Philippines. Findings from this study shall be useful in crafting equine tick and TBD control and prevention programs in the country.

## 1. Introduction

Tick-borne diseases (TBDs) are among the major hindrances to the global livestock industry. In horses, common tick-borne infections are caused by protozoan *Babesia caballi* and *Theileria equi*, and by the zoonotic bacteria *Anaplasma phagocytophilum, Borrelia burgdorferi* sensu lato, *Rickettsia*, and *Coxiella burnetii* [1,2,3,4,5].

Equine piroplasmosis (EP) is a debilitating disease caused by piroplasm parasites *B. caballi*, *T. equi*, and *T. haneyi*, affecting equids, mainly horses, mules, donkeys, and zebras, worldwide [1]. The geographic distribution of EP corresponds to that of its tick vectors, with only a handful of countries regarded as non-endemic [6]. There are four and six genera of hard ticks implicated as competent vectors for *B. caballi* and *T. equi* group, respectively [7]. Clinical signs associated with acute *B. caballi* and *T. equi* infections may include fever, anorexia, weight loss, malaise, dehydration, tachycardia, edema, anemia, icterus, and hemoglobinuria, and severe cases may result in mortalities [8,9], while *T. haneyi* infection is clinically inapparent [10]. Aside from the adverse impacts on health, the hampering of equine international trade is a major issue imposed by EP [11]. 

The cosmopolitan bacteria *A. phagocytophilum* is the causative agent of ruminant tick-borne fever and of granulocytic anaplasmosis in a multitude of hosts, i.e., equines, canines, felines, bovines, and humans [2]. Most of the clinical signs of equine granulocytic anaplasmosis (EGA) are non-specific, but a defining hematologic abnormality is the occurrence of thrombocytopenia [12]. Severe cases of EGA can be fatal [13]. Majority of the EGA reports are from Europe, Canada, and the USA [14].

Borreliosis is a zoonotic disease caused by infection with *B. burgdorferi* sensu lato (Bbsl) spirochetes, which are transmitted through the bites of infected *Ixodes* ticks. The Bbsl complex, composed of 21 species [15], is maintained in the wild through small mammals such as rodents, while humans and domesticated vertebrates, such as cattle, dogs, and horses, are some of its incidental hosts [16]. Typical clinical attributes of borreliosis in horses may include neuroborreliosis, uveitis, and cutaneous pseudolymphoma, although definitive diagnosis for equine borreliosis can be complicated [3]. In humans, Lyme borreliosis is widespread in the northern hemisphere countries and is an epidemic [3,17,18]. Various genospecies of Bbsl have been detected in different tick species, animals, and humans in several East and Southeast Asian countries such as Japan [19], China [19], Taiwan [19], Thailand [20,21], and Malaysia [22,23].

Diseases caused by *Rickettsia* and *C. burnetii* are also potentially zoonotic. Several *Rickettsia* species have been detected in tick vectors. Still, only a few species, specifically *Rickettsia rickettsii*, *R. felis*, *R. raoultii*, and *R. slovaca* have been molecularly detected in horses from other countries [24,25], with *R. rickettsii* recently linked to a clinical case [4]. On the other hand, horses are considered potential reservoirs of *C. burnetii*, the etiological agent of Q fever [5]. In recent years, *C. burnetii* in horses was molecularly documented in Asian countries such as China [25], South Korea [26], and Iran [27]. 

In the Philippines, the estimated 252,000 heads of horses are primarily used for racing, transportation, and meat production [28]. Of these, horse racing is considered the most lucrative, with an annual contribution of approximately 26 million USD [29]. In previous studies, the presence of *B. caballi* and *T. equi* parasites infecting horses were confirmed by microscopy [30], while both parasites were detected in horses using polymerase chain reaction (PCR) assays [31]. Similarly, both studies demonstrated varying exposure of horses to *B. caballi* and *T. equi* using immunochromatographic test strips [30,31]. Given EP’s enormous economic impacts, surveillance is required to prevent potential outbreaks, especially in areas where EP-naïve horses are introduced regularly, such as in race parks. To the authors’ knowledge, there has been no report on equine borreliosis, EGA, rickettsiosis, and Q fever or the detection of their respective causative agents in Philippine horses. Therefore, the present study aimed to identify selected tick-borne pathogens (TBPs) through molecular assays and determine the risk factors associated with TBP infections in thoroughbred racehorses in the Philippines.

## 2. Results

### 2.1. Sample Population and Demographics

**Figure 1:**
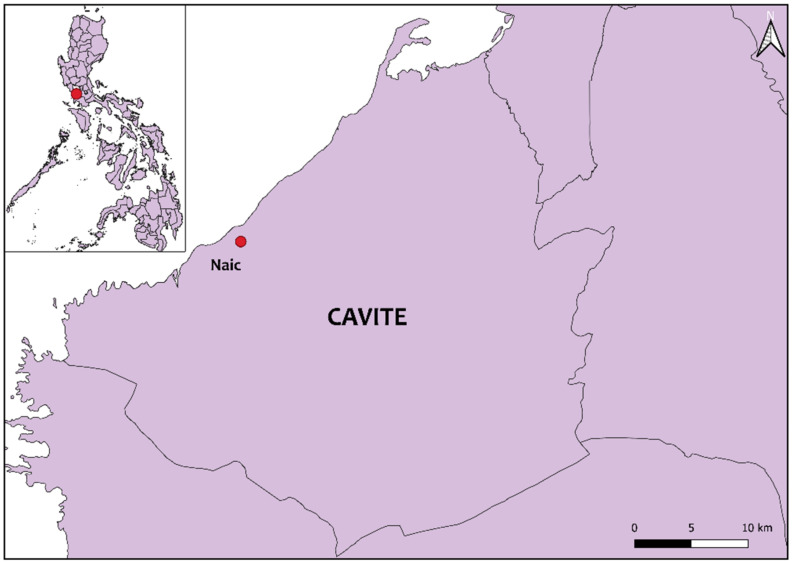
Location map of the sampling site in Cavite province, Philippines. The Philippine map, with the sampling site indicated in red, is shown in the inset.

We sampled a total of 124 (n = 124) horses raised in a race park in Cavite, Philippines (Figure 1). All horses were thoroughbreds and were specifically raised for racing. The majority of the horses were female (56.45%), while the age of the horses ranged from 1 to 20 years (median = 5). The sample population was composed of 12.90% (16/124) yearlings, 17.74% (22/124) colt/filly, and 69.35% (86/124) stallion/mare (Table 1). During sample collection, ticks and other ectoparasites were not observed in the bodies of the horses. The caretakers reported that ectoparasite control practices included daily bathing of the horses and the use of ducks as biological means of controlling ectoparasites. Moreover, none of the sampled horses presented any clinical signs related to TBDs.

### 2.2. Detected Tick-Borne Pathogens 

Of the 124 screened samples, at least one pathogen was confirmed in 48.39% (n = 60) (Table 2). *Babesia caballi*, *T. equi*, *A. phagocytophilum*, *A. marginale*, Bbsl, and *C. burnetii* were the TBPs detected in 12.10% (n = 15), 0.81% (n = 1), 10.48% (n = 13), 0.81% (n = 1), 38.71% (n = 48), and 0.81% (n = 1) of the samples, respectively (Table 1). The positive samples showed distinct bands which corresponded to the expected target sizes for *B. caballi* (~540 bp), *T. equi* (~392 bp), *A. phagocytophilum* (~928 bp), *A. marginale* (~768 bp), Bbsl (226–266 bp), and *C. burnetii* (~1450 bp). Conversely, all samples were negative for *Rickettsia* (Table 1). Forty-two samples had single infections while 18 samples were coinfected with two or more TBPs (Table 2). Concurrent infection of *B. caballi* and Bbsl and *A. phagocytophilum* and Bbsl were recorded as the two most frequent coinfection types.

### 2.3. Identifying Risk Factors Associated with TBP Detection

Univariable analysis by Pearson’s chi-squared or Fisher’s exact test was done to assess the association of PCR-positivity with the different variables (Table 1 and Table 3). Samples positive for *T. equi*, *A. marginale*, and *C. burnetii* were excluded in the analysis due to low detection rates. Female horses had significantly higher *B. caballi* infection (18.57%) compared to those of male horses (3.70%) (*p* = 0.013). Gender was negligible in *A. phagocytophilum* (*p =* 0.77) and Bbsl (*p* = 0.58) infections. On the other hand, *B. caballi* (25%) and *A. phagocytophilum* (18.75%) were more frequently detected in yearlings, while Bbsl detection rates were comparable among various age groups. Nevertheless, the differences among age groups were not significant, as shown by *p* values of 0.17, 0.44, and 0.96, respectively.

Multivariable logistic regression analysis was performed for *B. caballi*, *A. phagocytophilum*, and Bbsl (Table 4). Variables that had *p* values ≤ 0.20 for *B. caballi* (gender: *p* = 0.013; age group: *p* = 0.17; Bbsl positivity: *p* = 0.09), *A. phagocytophilum* (Bbsl positivity: *p* = 0.13), and Bbsl (*B. caballi* positivity: *p* = 0.09; *A. phagocytophilum* positivity: *p* = 0.13) in the univariable analysis were included in the subsequent methods. Using the backward stepwise method, the *B. caballi* model, which included gender and Bbsl positivity as predictors, showed the lowest Akaike information criterion (AIC) value (87.54); thus, it was selected as the final model. Analysis indicated that gender was a significant risk factor for *B. caballi* infection (*p* = 0.026), with female horses 5.77 times more likely to test positive for *B. caballi* than male horses (Table 4). Meanwhile, the final models for the other TBPs identified Bbsl positivity, and *B. caballi* and *A.phagocytophilum* positivity as non-significant predictors for detecting *A. phagocytophilum* and Bbsl in horse samples, respectively (Table 4).

### 2.4. Sequence Identities and Phylogenetic Analysis

**Figure 2:**
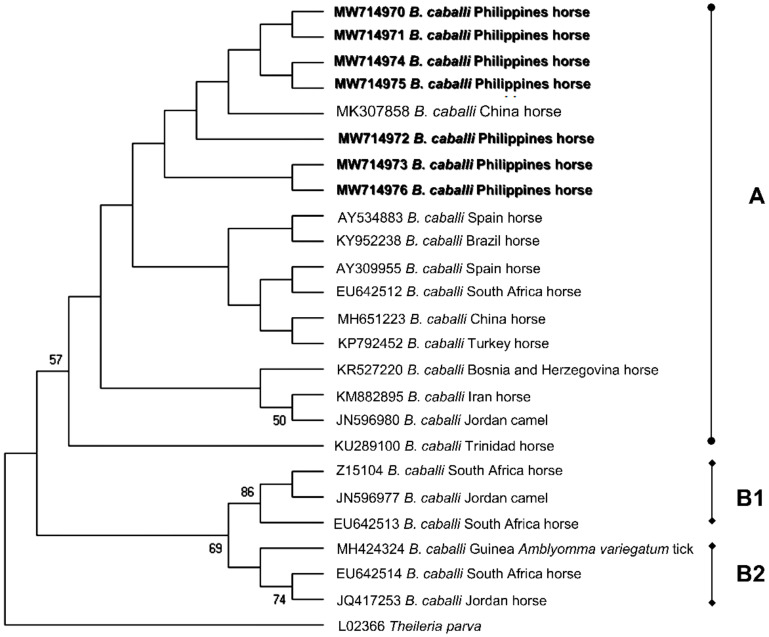
Phylogenetic analysis of *B. caballi* based on the 18S rRNA gene. The phylogeny was inferred using the maximum likelihood method and Hasegawa-Kishino-Yano model with a discrete Gamma distribution (+G, parameter = 0.3348). The phylogeny test used was the bootstrap method with 1000 replications. The sequences obtained from the current study are shown in bold. *Theileria parva* was used as an outgroup.

**Figure 3:**
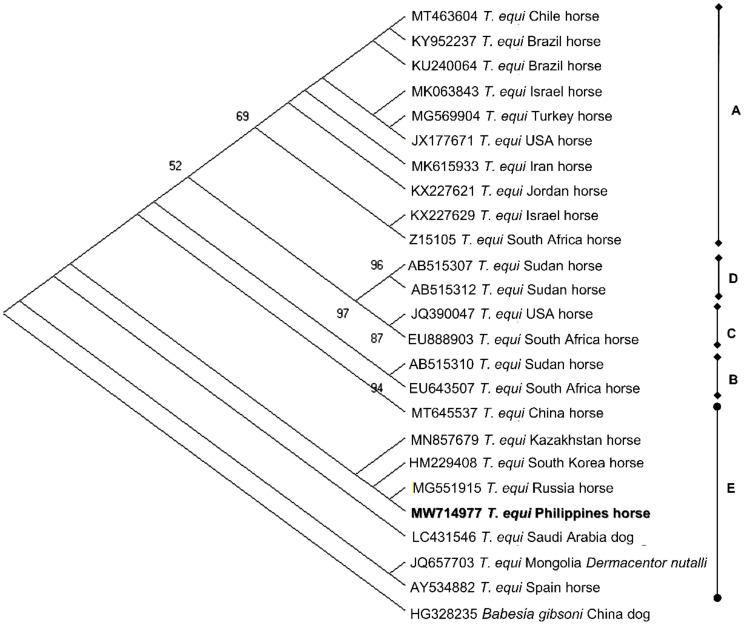
Phylogenetic analysis of *T. equi* based on the 18S rRNA gene. The phylogeny was inferred using the maximum likelihood method and Tamura-3 parameter model with a discrete Gamma distribution (+G, parameter = 0.3320). The phylogeny test used was the bootstrap method with 1000 replications. The sequence obtained from the current study is shown in bold. *Babesia gibsoni* was used as an outgroup.

Randomly selected amplicons were sequenced to obtain representative sequences for *B. caballi* (n = 7), *T. equi* (n = 1), *A. phagocytophilum* (n = 6), and Bbsl (n = 17). *Babesia caballi* 18S rRNA isolates from the current study (MW714970-MW714976) shared 98.63–100% identity with each other and were 99.15–100% identical to a *B. caballi* isolate from a horse in China. Phylogenetic analysis indicated that the predominant genotype of the *B. caballi* population in racehorses in Cavite, Philippines was genotype A and these sequences were most closely related to a *B. caballi* isolate from China (Figure 2). On the other hand, the sole *T. equi* sequence (MW714977) had the highest shared percent identity (99.77%) with *T. equi* 18S rRNA isolates from China, Kazakhstan, Russia, Mongolia, Saudi Arabia, South Korea, and Spain. The phylogeny indicates that MW714977 is classified as genotype E and formed a sister clade with dog and horse *T. equi* isolates from Saudi Arabia and South Korea, respectively (Figure 3).

**Figure 4:**
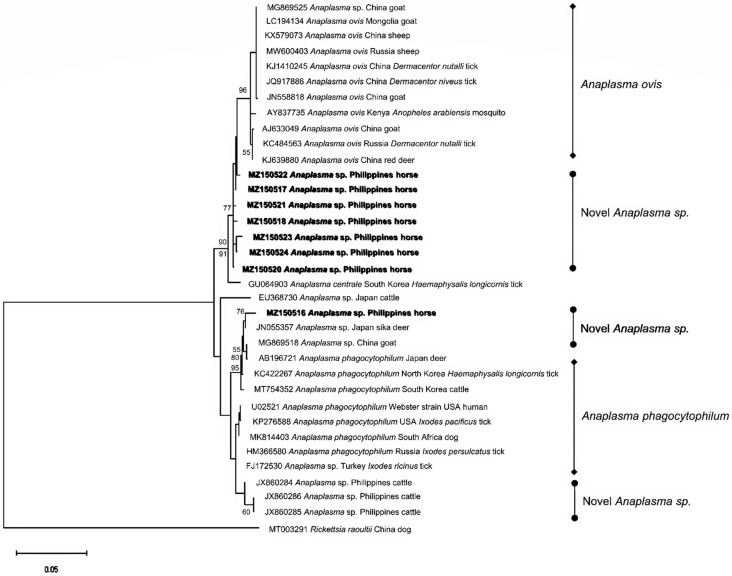
Phylogenetic analysis of *Anaplasma* spp. based on the 16S rRNA gene. The phylogeny was inferred using the maximum likelihood method and Hasegawa-Kishino-Yano model with a discrete Gamma distribution (+G, parameter = 0.2268). The phylogeny test used was the bootstrap method with 1000 replications. The sequences obtained from the current study are shown in bold. *Rickettsia raoultii* was used as an outgroup.

*Anaplasma* 16S rRNA sequences from eight distinct genotypes were obtained from six PCR-positive samples (MZ150516-MZ150518; MZ150520-MZ150524). MZ150516 was most closely related (99.03%) to a novel *Anaplasma* sp. (*A. phagocytophilum*-like) isolate from a sika deer in Japan (JN055357). Meanwhile, the remaining sequences (n = 7) shared 98.81–99.68% identity with each other and had >99% identities with *A. ovis* isolates from China, Mongolia, Russia, and Kenya. Phylogeny inference revealed the grouping of the *Anaplasma* sp. (*A. ovis*-like) lineages generated in the present study, deviating from the *A. ovis* clade. Additionally, MZ150523 and MZ150524 formed a well-supported subclade within the aforementioned group (Figure 4). The alignment analysis indicated that the *A.ovis*-like variant MZ150523 had nine single nucleotide polymorphisms (SNPs) compared to *A. ovis* sequences from Russia (MW600403), China (KJ639880), and a horse isolate (MZ150522) from this study (Figure S1). On the other hand, MZ150516 clustered with other *A. phagocytophilum*-like isolates from East Asian countries and clearly diverged from the main *A. phagocytophilum*-like clade, forming a phylogenetically distinct subclade (Figure 4). Moreover, 21 SNPs and two deletions were found by the alignment analysis of MZ150516 with U02521, the Webster strain of *A. phagocytophilum* (Figure S2). The lone *A. marginale groEL* sequence (MZ408312) was 100% identical to previous isolates from the Philippines, while several reamplification attempts of the *C. burnetii*-positive sample failed; hence, no sequence for *C. burnetii* was generated.

**Figure 5:**
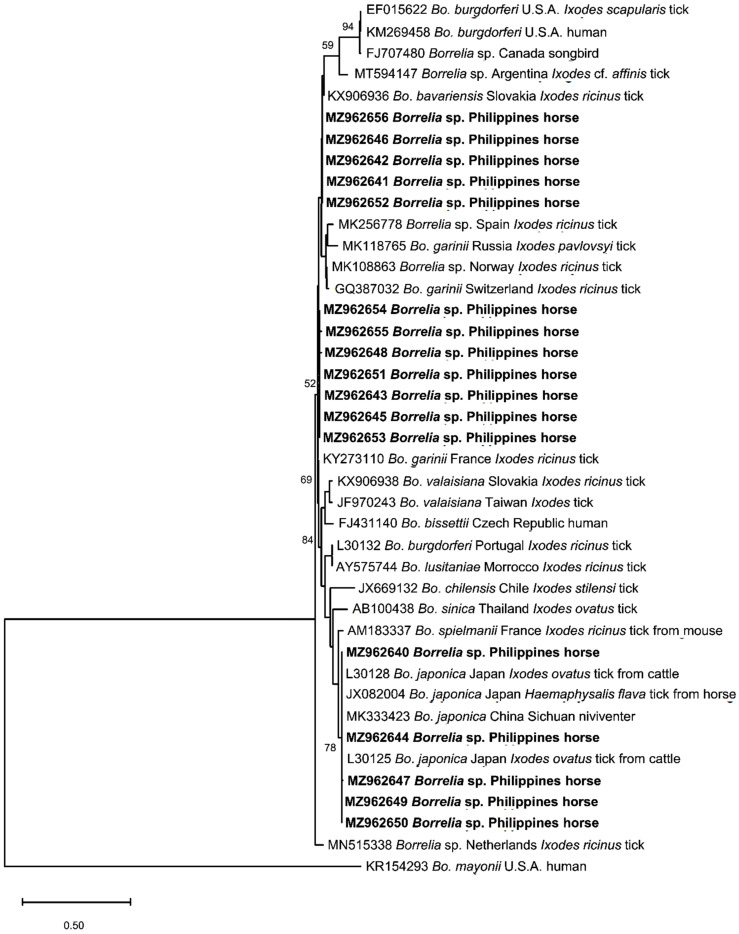
Phylogenetic analysis of *Borrelia* spp. based on the 5–23S rRNA intergenic spacer sequences. The phylogeny was inferred using the maximum likelihood method and Tamura-3 parameter model with a discrete Gamma distribution (+G, parameter = 0.6683). The phylogeny test used was the bootstrap method with 1000 replications. The sequences obtained from the current study are shown in bold. *Borrelia mayonii* was used as an outgroup.

Of the 17 *Borrelia* 5–23S rRNA intergenic spacer sequences, five isolates (MZ962640; MZ962644; MZ962647; MZ962649; MZ962650) were 98.73–100% identical with each other and showed high percent identity (99.36–100%) with *B. japonica* isolates from Japan and China, as confirmed in the phylogenetic analysis (Figure 5). Additionally, five sequences (MZ962641; MZ962642; MZ962646; MZ962652; MZ962656) and seven sequences (MZ962643; MZ962645; MZ962648; MZ962651; MZ962653- MZ962653) shared identities of 96.05–100% and 95.28–99.61%, respectively, and formed their own groups. These 12 isolates were most closely related to a Spanish *Borrelia* sp. (MK256778) obtained from *Ixodes ricinus* tick, with 98–98.4% similarity with the sequences in the former group and 95.3–96.5% similarity with the sequences in the latter group.

## 3. Discussion

The long-established horse racing industry is a huge income generator in the Philippines. Despite this, the equine TBD research in the Philippines remains scarce and has progressed only modestly in recent decades. In this study, we molecularly identified major agents of TBDs in the blood DNA samples of Philippine racehorses. 

EP is one of the most critical diseases causing devastating economic impacts to the equine industry [8,9]. The current study demonstrates the prevalence of EP in Philippine racehorses. The detection rates for *B. caballi* (12.10%) and *T. equi* (0.81%) in this study reflect the results of the previous EP investigation in slaughter and racehorses from Batangas and Manila, wherein *B. caballi* was the predominant EP agent identified [30]. It should be noted that the sampling site of the study conducted in 2010 in Manila transferred the horse racing operations to the present sampling location in Cavite. Conversely, *T. equi* was more frequently detected by PCR in horses in Cebu and Bohol provinces [31]. An in-country variance of the frequency of EP infections has been documented in several studies and was attributed to the differences in extrinsic factors such as geographic features [32,33], microclimate [34], and management practices of the animals [35]. The horses positive for EP did not exhibit any clinical sign associated with EP, a hallmark of persistent infections [7]. This type of infection is common in countries where EP is prevalent, such as the Philippines [6]. Inapparent carriers of EP pose a transmission risk as ticks can efficiently transmit the parasites acquired from persistently infected horses to naïve ones [36]. Moreover, EP carrier horses used for sports, such as racing, may have suboptimal performance compared with their healthy counterparts despite the lack of evident clinical signs [9]. Nevertheless, EP-carrying horses should be treated to eliminate parasites as developing overt clinical EP can still occur, especially in fatigued and immunosuppressed horses [37]. 

Identifying EP risk factors is vital to better tailor fit the formulation of control measures for EP in a particular horse population. Gender was identified as an important risk factor for *B. caballi* infection in this study. A higher positivity (18.37%) and higher odds (5.77) for *B. caballi* infection was recorded for female horses compared to that of male horses, which contradicts the findings of Qablan et al. [38]. However, previous investigations which evaluated gender as a risk factor for EP have inconsistent and contrasting results [1,39]. Although not significantly different, we likewise observed an indirect relationship between *B. caballi* infection rates and age. This can be attributed to the more robust immunity in older animals that leads to the elimination of *B. caballi* parasite [34,40], a feature of *B. caballi* persistent infections. 

Equine piroplasma genotyping based on the 18S rRNA gene has been extensively used to characterize parasite populations in the field [6]. This study’s identification of *T. equi* genotype E provides invaluable information on the *T. equi* population circulating in Philippine horses. This genotype was implicated in prior fatal cases of equine theileriosis [41] and from apparently healthy horses from China [42] and South Korea [43]. As we only sequenced one isolate, further study on *T. equi*-infected horses should be conducted in various provinces to fully elucidate the different types of existing *T. equi* populations in the Philippines. On the other hand, *B. caballi* genotyping is relatively limited, with isolates only classified into three genotypes [6]. We confirmed genotype A as the predominant *B. caballi* in the tested horses in the present survey. Unlike *T. equi*, *B. caballi* 18S rRNA genotypes reveal inadequate insight to the parasite’s geographical distribution. Thus, characterization using different gene markers shall be beneficial to fully characterize *B. caballi* populations in equines.

We herein report the first molecular identification of *Anaplasma* in horses in the Philippines. While *Anaplasma* is relatively more frequently reported in cattle and water buffaloes in the country [44], information about it in other Philippine livestock is lacking. For this survey, we initially aimed to detect *A. phagocytophilum*, the species responsible for EGA. Interestingly, sequencing the 16S rRNA gene confirmed the presence of novel variants of *Anaplasma* instead. The *Anaplasma* sequences generated in this study exhibited the same values for sequence similarity and percent identities (98.81–99.68% for *A. ovis*-like and 99.03% for *A. phagocytophilum*-like). The sequence similarity values fall within the cutoff values of 98.65% for species delineation for prokaryotes based on the 16S rRNA gene [45], indicating that these sequences are possibly new variants of *A. ovis* and *A. phagocytophilum*. This result reinforces the previously described high diversity of *Anaplasma* in the Philippines [46] and may indicate the expansion of *Anaplasma* species in various hosts.

The detection of *A. ovis*-like variants in horses is particularly noteworthy, as horses and goats are raised together inside the race park. The two animals were observed to interact very closely during the sampling. Cross-species transmission, with goats implicated as pathogen source, may be a plausible explanation for detecting *A*. *ovis*-like variants in horses in this study. Presently, the competency and host specificity of equine *Anaplasma* vectors in the Philippines has not been reported, and the status of *Anaplasma* infections in goats is not confirmed. Hence, verifying this premise will be helpful to confirm *Anaplasma* inter-host transmission and the clinical impact of *A. ovis*-like variant infection to its incidental hosts. On the other hand, an *A. phagocytophilum*-like variant was detected in this study. In a prior survey in Cebu province, the authors identified several *A. phagocytophilum*-like variants in cattle [47]. As *A. phagocytophilum* has a diverse host range [48], numerous strains and variants have evolved which considerably differ in their ability to cause diseases [49]. Notably, the phylogenetic placing of the identified *A. phagocytophilum*-like variant in a subclade with isolates from East Asia suggests a geographic-related grouping. Nonetheless, using a multi-locus characterization approach may provide a more comprehensive strain differentiation of *A. phagocytophilum* in horses [50]. 

The first molecular evidence of *Borrelia* in the Philippines is documented in the present study. The presence of *Borrelia* has never been confirmed in the Philippines before, and the detection of DNA fragments in horse samples may indicate possible non-clinical Bbsl infection. In particular, we detected the Bbsl genospecies *B. japonica*, which is vectored by *I. ovatus* ticks and thought to be a Japan-specific, non-pathogenic human Bbsl genospecies [19], and two *Borrelia* sp. genotypes closely related to a *Borrelia* isolate highly similar to *B. garinii*. Cases of horses acting as potential reservoir for Bbsl causing human disease have been documented in South Korea [51] and Belgium [52] before. Given its zoonotic nature, the detection of Bbsl in horses demands a more extensive inquiry into the disease transmission risk to humans who have close contact with the infected animals.

We detected one horse sample positive for *C. burnetii*. Recently, *C. burnetii* was molecularly detected in cattle, water buffaloes, and *Rhipicephalus microplus* ticks from nearby provinces of Rizal and Quezon [53] while sera from human and ruminants collected from Laguna, Northern Samar, and General Santos City were seropositive for *C. burnetii* [54]. On the contrary, *Rickettsia* was not detected in horse samples in the present study. Molecular studies of *Rickettsia* in the Philippines are limited. *Rickettsia* was recently identified in dogs in Luzon [55], but was not detected in ruminants and ticks [53]. However, non-detection of *Rickettsia* could not rule out probable infections as detecting this bacterium using PCR assays in whole blood DNA can be less sensitive than detection in DNA extracted from cutaneous and serum samples [56].

Despite the information on equine TBPs uncovered in the current study, the competent tick vectors of the detected TBPs remain unidentified. The tick species transmitting *B. caballi*, *T. equi*, and *A. ovis*-like variant have not been reported in Cavite province, but potential tick vectors, such as *Rhipicephalus microplus* and *Rhipicephalus sanguineus*, were previously identified from various animal hosts in nearby provinces [55,57,58]. In addition, the detection of an *A. phagocytophilum*-like variant and Bbsl in the current study should compel further investigation on the competent tick vectors of these potentially zoonotic pathogens. Ticks belonging to the *Ixodes* genus are the only confirmed competent vectors of *A. phagocytophilum*. Still, previous studies suggest an alternative epidemiological cycle in *Rhipicephalus* ticks [59], which may explain our results. Likewise, *Ixodes* ticks are the only confirmed competent tick vectors of *Borrelia* [15]. Detecting these *Ixodes*-transmitted pathogens is not unlikely, and the presence of *Ixodes* ticks in Cavite is highly probable, as a tick survey conducted 50 years ago identified *I. granulatus* ticks in wild rodents [60]. 

In addition, several matters are beyond the scope of our study. We recommend employing direct methods such as immunofluorescence microscopy and isolation of the organism, and serological confirmation of infection by antibody detection to address the various sensitivity issues of PCR assays for future studies. In particular, the relatively high detection rate for *Borrelia* in the current study may be complemented by serological confirmation, the more common and accepted diagnostic test for *Borrelia* infection [3,18]. 

Altogether, the information gathered from this molecular survey revealed the TBPs harbored by thoroughbred racehorses and the horses’ potential role as reservoirs of TBPs. Moreover, this study describes data on EP genotypes, novel variants of *Anaplasma*, and molecular evidence of *Borrelia* in Philippine racehorses for the first time.

## 4. Materials and Methods

### 4.1. Ethical Statements

The methodologies and procedures performed in this study were approved by Obihiro University of Agriculture and Veterinary Medicine, Obihiro, Hokkaido, Japan (animal experiment permit no. 20-128; DNA experiment permit no. 1723-4). Animal handling practices adhered to the Philippine Animal Welfare Act (Republic Act 8485 as amended by R.A. 10631) and were approved by the Institutional Animal Care and Use Committee (IACUC) of Cavite State University, Indang, Cavite, Philippines (approval no. 2019-001). The caretakers of the horses and the race park manager were informed about the background and aims of the study and gave their consent before sampling was conducted under the supervision of the race park veterinarian.

### 4.2. Location of Sampling and Animals

Cavite is a province situated southwest of Manila and located in the southern part of Luzon island, Philippines. The province is characterized by various topographies ranging from coastal plains to upland mountainous areas. Most areas in Cavite is suited for agricultural production, which is supported by its climate type composed of two pronounced seasons: the dry season from November to April and the rainy season from May to October [61]. Two of the three major racehorse parks in the Philippines are located in Cavite province. For this study, a total of 124 racehorses were randomly selected as samples from a race park in Naic, Cavite. Name, gender, age, breed, clinical signs, and history of tick infestation of each animal were recorded through the information provided by the caretakers. Briefly, about 3 ml of whole blood was drawn from the jugular vein and collected in Vacutainer^®^ EDTA tubes (BD, Franklin Lakes, NJ, USA). The blood samples were kept cool in an ice box during transportation to the laboratory. All samples were processed within the same day of the sampling.

### 4.3. Extraction of DNA

DNA was extracted using the QIAmp DNA Blood Mini Kit (Qiagen, Hilden, Germany), following the manufacturer’s protocol. About 200 μL of whole blood was used and DNA was eluted in 100 μL elution buffer. DNA concentration was checked using NanoDrop^™^ 2000 Spectrophotometer (Thermo Fisher Scientific, Waltham, MA, USA). Samples were stored at −20 °C until the molecular screening.

### 4.4. PCR Assays for the Identification of Tick-Borne Pathogens

Samples were screened using PCR assays specific to selected TBPs. A summarized list of information on the assays used in this study is shown in Table 5. A multiplex nested PCR assay which amplifies the partial 18S rRNA gene of *B. caballi* (540 bp) and *T. equi*/*T. haneyi* (392 bp) was used for detecting equine piroplasma [62]. Meanwhile, PCR assays for the detection of tick-borne bacteria *A. phagocytophilum* [63], *Rickettsia* spp. [64], and *C. burnetii* [65] amplified the partial 16S rRNA gene with final amplicon sizes of 928, 426, and 1450 bp, respectively. The rrf-rrl (5–23S rRNA intergenic spacer)-target primer set was used for the detection of Bbsl which amplified bands of 226–266 bp [66]. For the nested PCR assays, the first assay reactions were set to a volume of 10 µL with a final concentration of 2 mM of each dNTP (New England Biolabs, Ipswich, MA, USA), 1× ThermoPol^®^ buffer (New England Biolabs), 2 µM of forward and reverse primers, 0.25 U of Taq DNA polymerase (New England Biolabs). The second or single assays were performed to a final volume of 25 µL with the same final concentration of reagents except for the final concentrations of primers in the multiplex EP assay: forward primer (4 µM) and reverse primers (2 µM for *B. caballi* and 2 µM for *T. equi*/*T. haneyi*). About 2 µL of genomic DNA was used for direct amplification from the samples, while 1.5 µL of the first reaction product was used as a template for the multiplex or nested reactions. Thermocycling conditions were performed for each pathogen as before, except for the extension and final extension temperatures, which were set at 68 °C. In all assays, double-distilled water was used as negative controls and confirmed positive DNA samples from previous studies conducted in our laboratory were used as positive controls (Table 5). PCR products were resolved in either 1% or 1.5% agarose gel by electrophoresis, stained with ethidium bromide, and visualized under UV light.

### 4.5. Sequencing and Phylogenetic Analyses

Amplicons were excised from the gel and purified using NucleoSpin^®^ Gel and PCR Clean-up (Macherey Nagel, Düren, Germany). Samples with high concentration were directly sequenced; otherwise, samples with low concentration were ligated to pGEM^®^-T Easy Vector (Promega Corporation, Madison, WI, USA) and inserted in DH5α *Escherichia coli* calcium competent cells. Plasmids were purified using NucleoSpin^®^ Plasmid QuickPure Kit (Macherey Nagel). Purified products were sequenced using BigDye^™^ Terminator v3.1 Cycle Sequencing Kit (Applied Biosystems, Foster City, CA, USA) and ABI Prism 3100 Genetic Analyzer (Applied Biosystems). 

After manual trimming and assembly, sequences were submitted to the NCBI GenBank database. The accession numbers are: MW714970-MW714976 (*B. caballi* 18S rRNA, 575–584 bp); MW714977 (*T. equi* 18S rRNA, 435 bp); MZ150516-MZ150524 (*Anaplasma* sp. 16S rRNA, 924 bp); MZ408312 (*A. marginale groEL*, 855 bp); MZ962640- MZ962656 (*Borrelia* sp. 5-23S rRNA, 236-256 bp). Shared percent identities among each other and with sequences in the GenBank database were determined through EMBL’s Clustal Omega multiple sequence alignment [67] and Nucleotide BLAST search, respectively. Multiple sequences were aligned by Clustal W, and phylogeny was inferred using Molecular Evolutionary Genetics Analysis (MEGA) X [68]. The models were determined using the maximum likelihood method, and the phylogeny test was performed using the bootstrap method with 1000 replications.

### 4.6. Statistical Analysis

The data were comprised of animal parameters (age and gender) as independent variables and positivity for *B. caballi*, *A. phagocytophilum,* or Bbsl as dependent variables. For coinfections, positivity for the first and second TBP was considered independent and dependent variables. Meanwhile, data for other detected TBPs were excluded in the case of low detection rates. Age was classified into three categories: yearling (≤2 years), colt/filly (<4 years), or stallion/mare (≥4 years), while gender was categorized as either male or female. Univariable analysis by Pearson’s chi-squared or Fisher’s exact test was performed to assess the association of PCR-positivity with the parameters (age group and gender) and positivity to another pathogen. Consequently, multivariable logistic regression was performed for parameters with *p* value of ≤0.20. Initially, variable selection using the backward stepwise elimination was performed. Then, the models with the lowest AIC values were chosen. A *p* value of <0.05 was considered significant. All analyses were done using the Stats [69] and Epitools [70] packages in R.

## 5. Conclusions

In this survey, *B. caballi*, *T. equi*, *A. ovis*-like, *A. phagocytophilum*-like, *A. marginale*, *Borrelia burgdorferi* sensu lato genospecies, and *C. burnetii* were molecularly detected in thoroughbred racehorses in Cavite, Philippines. In addition, gender was determined as a significant risk factor for *B. caballi* infection. Furthermore, we performed the first genotyping of EP agents in Philippine horses. These findings provide crucial information on the TBD agents and call for establishing and implementing strategic treatment and control programs for the neglected tick-borne infections in Philippine horses. 

## Figures and Tables

**Table 1:** Detection of TBPs and univariable analysis based on age group and gender of the horses.

Variable	n	*Babesia caballi*				*Anaplasma phagocytophilum*				*Borrelia burgdorferi* Sensu Lato				*Theileria equi (%)*	*Anaplasma marginale (%)*	*Coxiella burnetii (%)*	*Rickettsia* spp. (%)
		No. of Positives (%)	OR	95% CI	*p* Value	No. of Positives (%)	OR	95% CI	*p* Value	No. of Positives (%)	OR	95% CI	*p* Value				
**Age Group**																	
Yearling (≤2 years)	16	4 (25.00)	Ref.		0.17 ^#^	3 (18.75)	Ref.		0.44	6 (37.50)	Ref.		0.96	n.d.	n.d.	n.d.	n.d.
Colt/Filly (<4 years)	22	3 (13.64)	0.48	0.06–3.41		1 (4.55)	0.22	0.0038–3.00		9 (40.91)	1.15	0.26–5.37		n.d.	n.d.	1 (4.55)	n.d.
Stallion/Mare (≥4 years)	86	8 (9.30)	0.31	0.07–1.64		9 (10.47)	0.51	0.11–3.31		33 (38.37)	1.04	0.31–3.82		1 (1.16)	1 (1.16)	n.d.	n.d.
**Gender**																	n.d.
Male	54	2 (3.70)	Ref.		0.013 ^#,^*	5 (9.26)	Ref.		0.77	19 (35.19)	Ref.		0.58	1 (1.85)	1 (1.85)	1 (1.85)	n.d.
Female	70	13 (18.57)	5.86	1.24–55.90		8 (11.43)	1.26	0.34–5.23		29 (41.43)	1.30	0.59–2.91		n.d.	n.d.	n.d.	n.d.
**Total**	124	15 (12.10)				13 (10.48)				48 (38.71)				1 (0.81)	1 (0.81)	1 (0.81)	n.d.

* A *p* value < 0.05 was considered significant. ^#^ Variables with *p* value ≤ 0.20 were included in the multivariable analysis. Abbreviations—OR: Odds ratio; CI: confidence intervals; Ref.: reference used; n.d.: not detected; No.: number.

**Table 2:** Infection type of TBP-positive samples.

Pathogen	Number of Positives	%
**Single infection**	42	33.87
*Babesia caballi*	5	4.03
*Theileria equi*	1	0.81
*Anaplasma phagocytophilum*	4	3.23
*Anaplasma marginale*	1	0.81
*Borreliaburgdorferi* sensu lato (Bbsl)	31	25
**Multiple infections**	18	14.52
Bbsl and *B. caballi*	9	7.26
Bbsl and *A. phagocytophilum*	7	5.65
*B. caballi* and *A. phagocytophilum*	1	0.81
Bbsl, *A. phagocytophilum*, and *C. burnetii*	1	0.81
**Total number of of positive samples**	60	48.39

**Table 3:** Univariable analysis of coinfection with two pathogens.

Coinfecting Pathogens	No. of Positives (%)	OR	95% CI	*p* Value
Bbsl and *B. caballi*	9 (7.26)	2.67	0.78–9.84	0.09 ^#^
Bbsl and *A. phagocytophilum*	8 (6.45)	2.81	0.75–11.71	0.13 ^#^
*B. caballi* and *A. phagocytophilum*	1 (0.81)	0.58	0.013–4.53	0.61

^#^ Variables with *p* value ≤ 0.20 were included in the multivariable analysis. Abbreviations—OR: Odds ratio; CI: confidence intervals; Bbsl: *Borrelia burgdorferi* sensu lato; No.: number.

**Table 4:** Multivariable logistic regression analysis of the identified risk factors.

Pathogen	Variable	Category	β	SE	*p* Value	OR	95% CI	Final Model AIC
*B. caballi*	Gender	Male	Ref.					87.54
		Female	1.75	0.79	0.026 *	5.77	1.23–27.03	
	Bbsl positivity	Negative	Ref.					
		Positive	0.95	0.58	0.10	2.58	0.83–8.03	
*A. phagocytophilum*	Bbsl positivity	Negative	Ref.					84.13
		Positive	1.04	0.6	0.08	2.84	0.87–9.27	
*Borreliaburgdorferi* sensu lato (Bbsl)	*B. caballi* positivity	Negative	Ref.					164.88
		Positive	1.06	0.57	0.063	2.89	0.95–8.82	
	*A. phagocytophilum* positivity	Negative	Ref.					
		Positive	1.12	0.61	0.067	3.06	0.93–10.12	

* A *p* value < 0.05 was considered significant. Abbreviations—β: regression coefficient; SE: standard error; OR: odds ratio; CI: confidence intervals; AIC: Akaike information criterion; Ref.: reference used.

**Table 5:** List of primer sets and PCR assay conditions used for detecting TBPs in racehorse samples.

Pathogen	Target Gene	PCR Assay Type	Primer Sequence (5′–3′)	Ta (°C)	Amplicon Size (bp)	Detection Threshold	Positive Control	Reference
Equine piroplasma	18S rRNA	multiplex-nested	GTTGATCCTGCCAGTAGTCA	54	913/867		*B. caballi*- and *T. equi*-positive horse gDNA	[62]
			CGGTATCTGATCGTCTTCGA					
			TCGAAGACGATCAGATACCGTCG	54				
*Babesia caballi*			CTCGTTCATGATTTAGAATTGCT		540	0.18 parasite cells		
*Theileria equi/Theileria haneyi*			TGCCTTAAACTTCCTTGCGAT		392	0.018 parasite cells		
*Anaplasma phagocytophilum*	16S rRNA	nested	TCCTGGCTCAGAACGAACGCTGGCGGC	50	1433	3 infected neutrophils	*A. phagocytophilum*-positive cattle gDNA	[63]
			AGTCACTGACCCAACCTTAAATGGCTG					
			GTCGAACGGATTATTCTTTATAGCTTGC	50	928			
			CCCTTCCGTTAAGAAGGATCTAATCTCC					
*Anaplasma marginale*	*groEL*	nested-touchdown	TCCTGGCTCAGAACGAACGCTGGCGGC	74–65	866	2 gene copies	*A. marginale*-positive cattle gDNA	[46]
			AGTCACTGACCCAACCTTAAATGGCTG					
			GTCGAACGGATTATTCTTTATAGCTTGC	74–68	618–768			
			CCCTTCCGTTAAGAAGGATCTAATCTCC					
*Rickettsia* spp.	16S rRNA	single	AACGTCATTATCTTCCTTGC	59	426	n.a.	*R. slovaca*-positive horse gDNA	[64]
			AGAGTTTGATCCTGGCTCAG					
*Coxiella burnetii*	16S rRNA	touchdown	ATTGAAGAGTTTGATTCTGG	58–48	~1450	n.a.	*C. burnetii*-positive horse gDNA	[65]
			CGGCTTCCCGAAGGTTAG					
*Borrelia burgdorferi* sensu lato	rrf-rrl (5–23S rRNA intergenic spacer)	nested	CGACCTTCTTCGCCTTAAAGC	57.6	412	n.a.	*B. garinii*-positive tick gDNA	[66]
			TAAGCTGACTAATACTAATTACCC					
			CTGCGAGTTCGCGGGAGA	55	226–266			
			TCCTAGGCATTCACCATA					

PCR: polymerase chain reaction; Ta: Annealing temperature; gDNA: genomic DNA; n.a. not available.

## Data Availability

The datasets generated during and/or analyzed during the current study are available from the corresponding author on reasonable request.

## Supplementary Materials

The following are available online at https://www.mdpi.com/article/10.3390/pathogens10101318/s1, Figure S1: Multiple sequence alignment analysis of *A. ovis*-like 16S rRNA sequences using Clustal Omega in EMBL-EBI. Sequences obtained from the current study (MZ150522 and MZ150523) were aligned with *A. ovis* sequences obtained from Russia (MW600403) and China (KJ639880). Purple boxes indicate single nucleotide polymorphisms (SNPs) in MZ150522, while red boxes display the SNPs in MZ150523. The primers are highlighted in yellow. Figure S2. Multiple sequence alignment analysis of *A. phagocytophilum*-like 16S rRNA sequence using Clustal Omega in EMBL-EBI. The sequence obtained from the current study (MZ150516) was aligned with *A. phagocytophilum* Webster strain obtained from the USA (U02521) and *A. phagocytophilum*-like sequence from Japan (JN055357). Purple boxes indicate single nucleotide polymorphisms (SNPs) and indels with reference to U02521 only, while red boxes display the SNPs and indels with reference to both U02521 and JN055357. The primers are highlighted in yellow.
