# Supplementary figures and images for: Molecular Identification of Selected Tick-Borne Protozoan and Bacterial Pathogens in Thoroughbred Racehorses in Cavite, Philippines
Source: Pathogens. 2021 Oct 13;10(10):1318. doi: 10.3390/pathogens10101318 (PMC8537292; doi:10.3390/pathogens10101318)

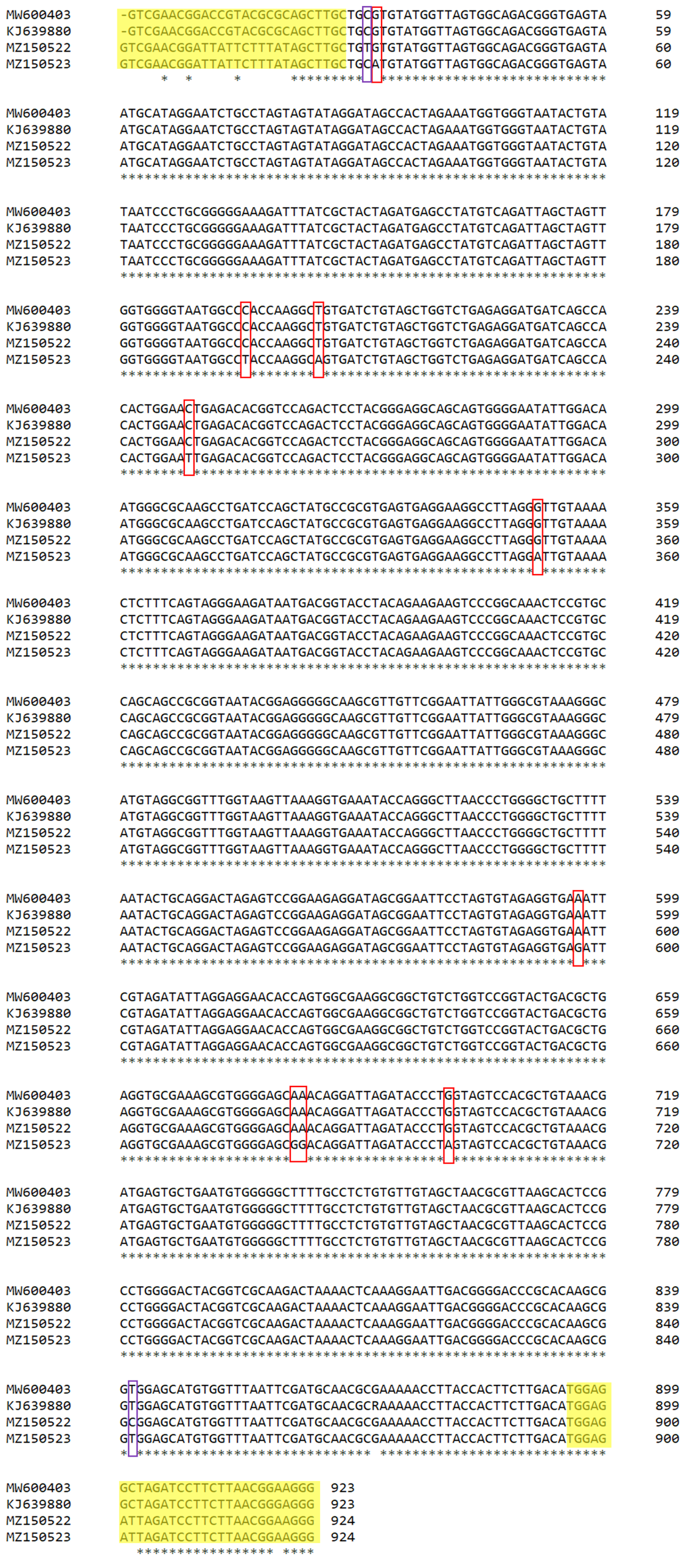

Supplement: Supplementary file 1 [file pathogens-10-01318-s001.zip › Supplementary Figure 1.tif]

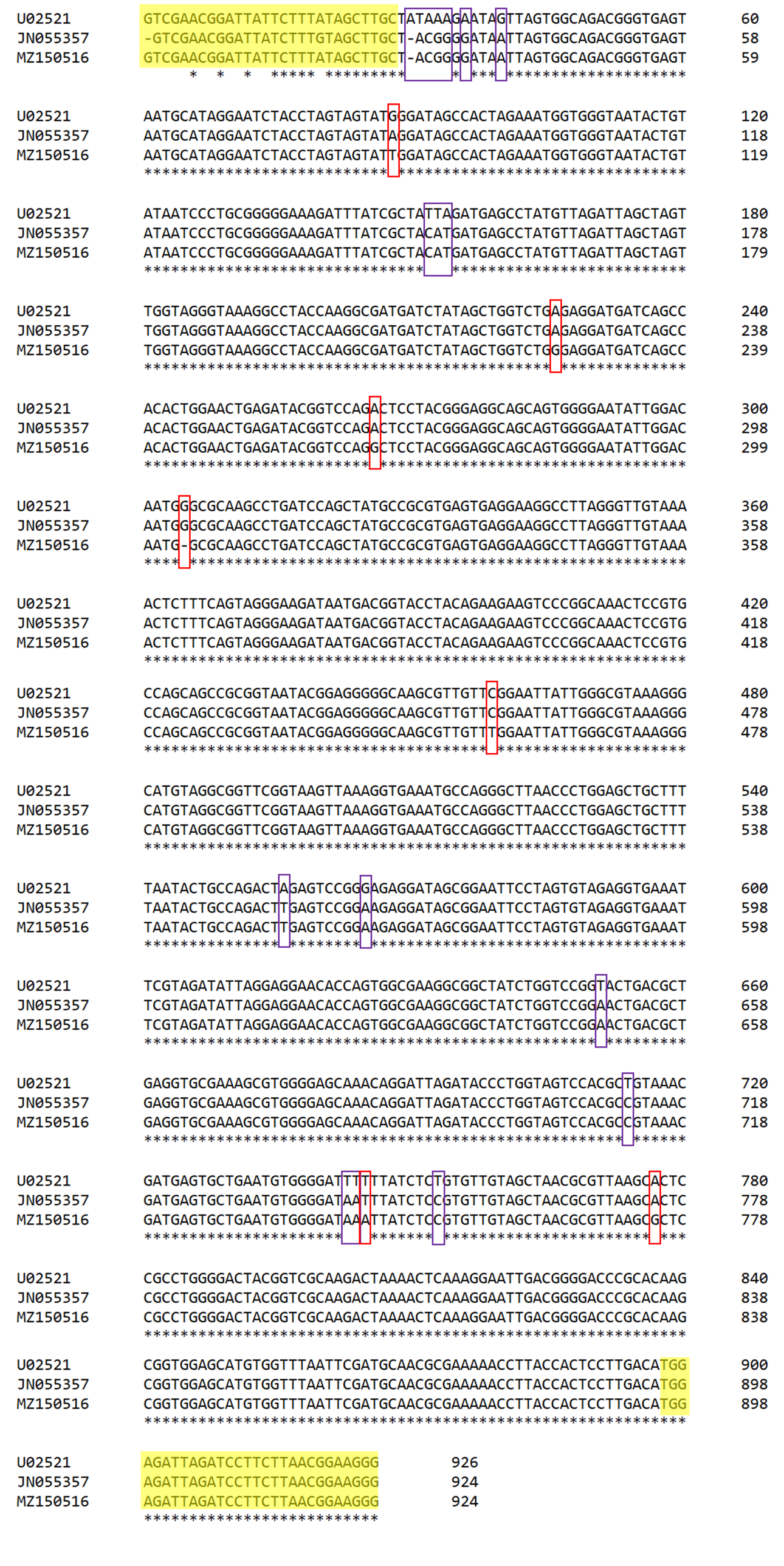

Supplement: Supplementary file 1 [file pathogens-10-01318-s001.zip › Supplementary Figure 2.tif]
